# The Coumarin Derivative 5′-Hydroxy Auraptene Suppresses Osteoclast Differentiation via Inhibiting MAPK and c-Fos/NFATc1 Pathways

**DOI:** 10.1155/2019/9395146

**Published:** 2019-12-28

**Authors:** Basem M. Abdallah, Enas M. Ali, Hany Elsawy, Gehan M. Badr, Ashraf M. Abdel-Moneim, Abdullah M. Alzahrani

**Affiliations:** ^1^Biological Sciences Department, College of Science, King Faisal University, Hofuf, Saudi Arabia; ^2^Endocrine Research (KMEB), Department of Endocrinology, Odense University Hospital and University of Southern Denmark, Odense, Denmark; ^3^Department of Botany and Microbiology, Faculty of Science, Cairo University, Cairo, Egypt; ^4^Department of Chemistry, Faculty of Science, King Faisal University, Hofuf, Saudi Arabia; ^5^Department of Chemistry, Faculty of Science, Tanta University, Tanta, Egypt; ^6^Department of Zoology, Faculty of Science, Ain Shams University, Cairo, Egypt; ^7^Department of Zoology, Faculty of Science, Alexandria University, Alexandria, Egypt

## Abstract

The phytochemical substances, coumarin derivatives, have demonstrated antiresorptive bone effects by suppressing osteoclast differentiation *in vitro* and *in vivo*. Recently, we have identified 5′-hydroxy auraptene (5′-HA), a coumarin derivative isolated from *Lotus lalambensis* Schweinf, as a novel stimulator for osteoblast differentiation. In this study, we investigated the effect of 5′-HA on osteoclast differentiation of mouse bone marrow (BM) cells. The effect of 5′-HA on BM cell proliferation and osteoclast differentiation was determined by measuring cell viability and tartrate-resistant acid phosphatase (TRAP) enzyme activity, quantification of TRAP^+^ multinucleated cells (TRAP^+^MNCs), and quantitative real-time PCR (qPCR) of osteoclastic gene expression. Regulation of NF-*κ*B, c-Fos/NFATc1, and MAPK signaling pathways by 5′-HA during osteoclastogenesis was measured by the NF-*κ*B reporter assay and Western blot analysis. 5′-HA significantly suppresses the receptor activator of NF-*κ*B ligand (RANKL) induced osteoclast differentiation of BM cells in a dose-dependent manner. Consistently, treatment of BM cells with 5′-HA significantly inhibited RANKL-induced activation of NF-*κ*B and c-Fos/NFATc1 pathways in a dose-dependent manner. Furthermore, RANKL-induced phosphorylation of ERK1/2, p-38, and JNK was significantly inhibited by 5′-HA in BM cells. In conclusion, we identified 5′-HA as a novel coumarin derivative that suppresses RANKL-induced osteoclastogenesis via inhibiting c-Fos/NFATc1 and MAPK signaling pathways.

## 1. Introduction

Osteoporosis is an endocrine-metabolic bone disease that is characterized by disorders in the bone remodeling process toward increased bone resorption by osteoclasts over the expenses of bone formation by osteoblasts [1]. Reduced bone mass and strength in osteoporotic patients' resulted in increased susceptibility of bone fractures, which is a major health problem in the elderly population [2, 3].

Osteoclasts are multinucleated cells that are derived from myeloid precursors upon the stimulation with cytokines, macrophage colony stimulating factor (MCS-F), and RANKL that are secreted by osteoblast lineage [4, 5].

The interaction between RANKL and osteoclast precursor cell surface RANK receptors triggers the signals of osteoclast differentiation via NF-*κ*B and MAPK pathways, which in turn recruit tumor necrosis factor (TNF) and receptor-associated factors and activate downstream signaling of the nuclear factor of activated T cells, cytoplasmic 1 (NFATc1), a master regulator of osteoclast differentiation. NFATc1 plays a vital role in osteoclast maturation and activation via upregulating the gene expression of osteoclastic-related genes including matrix metalloproteinase (*Mmps*), *Trap*, and Cathepsin K (*Ctsk*) [6–9]. Thus, targeting osteoclast formation and differentiation via inhibiting NF-*κ*B and MAPK signaling are an effective strategy toward preventing bone loss related diseases [10, 11].

In this context, several studies have demonstrated biological and pharmacological activities of plant-derived coumarin derivatives including antibacterial, antifungal, anti‐inflammatory, antioxidative, and antitumor effects [12–14]. Interestingly, numerous coumarin derivatives demonstrated *in vitro* and *in vivo* antiresorptive effects. Natural coumarin derivatives including daphnetin, psoralen, and wedelolactone were reported to exhibit an inhibitory effect on osteoclastic bone resorption [15, 16]. Furthermore, psoralidin, osthole, and aesculin were protective against bone loss in osteoporotic mouse models [17–19]. Recently, we identified a coumarin derivative, 5′-HA ([7-(5-hydroxy-3,7-dimethylocta-2,6-dienyloxy)-chromen-2-one]), as a novel compound that functions to stimulate osteoblast differentiation from BM-derived stromal stem cells in BMP-dependent mechanism [20]. To provide more detailed information on the effect of 5′-HA on bone metabolism, we aimed in this study to investigate the effect of 5′-HA on osteoclast differentiation of murine BM cells as well as to elucidate its molecular mechanism.

## 2. Materials and Methods

### 2.1. Extraction and Purification of 5′-HA

5′-HA was extracted from *Lotus lalambensis* Schweinf (collected from Saudi Arabia).

The extraction and identification of 5′-HA were performed as described previously by our group [20].

### 2.2. Osteoclast Culture

Bone marrow (BM) cells were isolated from 8-week-old male C57BL/6J mice as described previously [21]. Mice were bred and housed at the animal housing unit (College of Science, King Faisal University, Saudi Arabia) under standard conditions (21°C, 55% relative humidity) on a 12-hour light/12-hour dark cycle, and ad libitum food (Altromin® Spezialfutter GmbH & Co., Germany) and water were provided in accordance with the ethical clearance of the Standing Committee on Research Ethics. Mice were sacrificed by cervical dislocation and BM was flushed out from tibia and femur. BM was centrifuged for 1 min at 400 g and filtrated through a 70 *μ*m nylon mesh filter. BM cells were then plated in 96‐well plates at a density of 1 × 10^6^ cells/well in osteoclast differentiation medium (ODM) containing *α*‐MEM (Gibco BRL, Carlsbad, CA, USA) supplemented with 10% FBS (Gibco BRL), 100 U/mL of penicillin (Gibco BRL), 100 *μ*g/mL of streptomycin (Gibco BRL), 25 ng/mL of recombinant M‐CSF (R&D Systems, Minneapolis, MN, USA), and 25 ng/mL of recombinant RANKL (Pepro‐Tech, Rocky Hill, NJ, USA) to induce osteoclast formation. Cells were maintained at 37°C in a 5% CO_2_ incubator, and the medium was changed every 3 days.

### 2.3. Cell Viability Assay

BM cells were cultured in 96-well plates and then treated with different concentrations of 5′-HA for 3 days in the presence or the absence of RANKL and MCS-F. Cell viability was determined using the CellTiter-Blue® cell viability assay according to the manufacturer's instructions (Promega, USA) at OD 579.

### 2.4. Tartrate‐Resistant Acid Phosphatase (TRAP) Staining

BM cells were plated in 96-well plates and induced to osteoclast differentiation as described above. TRAP staining was performed at different time points according to the manufacturer's instruction for using the acid phosphatase, leukocyte (TRAP) kit (Sigma-Aldrich, Germany). TRACP^+^ MNCs containing more than three nuclei were considered to be osteoclasts and were evaluated using a reflected light microscope.

### 2.5. Measurement of TRAP Enzyme Activity

BM cells were induced to osteoclasts as mentioned above. At different time points, cells were washed with PBS, lysed in 30 *μ*L 0.1% Triton X-100 for 10 min, and a substrate solution of 100 *μ*L of PNPP (2 g/L *p*-nitro-disodium phenylphosphate, 7.6 g/L sodium L-tartrate, pH 5.2) was added. Cells were then incubated at 37°C for 30 min, and the reaction was stopped by the addition of 1 M NaOH. TRACP enzyme activity was measured at 405 nm absorbance in a microplate reader.

### 2.6. Osteoclast Bone Resorption Measurement

The activity of differentiated osteoclast was performed by the bone resorption assay kit (Cosmo Bio. Co. Ltd., Japan) according to the manufacturer's instructions. BM cells were cultured on fluoresceinated calcium phosphate-coated 24-well plates in the presence of M-CSF and RANKL without or with 5′-HA for 6 days. Fluorescent released from the calcium phosphate layer into conditioned medium due to osteoclast resorption activity was measured by detecting the fluorescence intensity at an emission wavelength of 535 nm.

### 2.7. Luciferase Reporter Assay

The modulation of NF-*κ*B pathway was determined by using Cignal™ NF-*κ*B luciferase Reporter Assay Kit (Qiagen Ltd., Manchester, UK). BM cells were cultured in 96-well plates and transfected with a mixture of NF-*κ*B luciferase reporter negative control or positive control, along with Renilla construct (as an internal control) according to the manufacturer's instructions using Lipofectamine 2000 (Thermo Fisher Scientific GmbH). Cells were induced with RANKL in the absence or the presence of different concentrations of 5′-HA and cultured for 24 h. Luciferase activities were determined using the Dual-Luciferase Assay System (Promega, Southampton, UK). Reporter activities were represented after normalization to the internal Renilla reporter.

### 2.8. Western Blot Assays

Cells were lysed at different time points in cell lysis buffer [22], supplemented with protease inhibitor cocktail (Roche Diagnostics, Mannheim, Germany). 30 *μ*g of protein was separated on 8% to 12% NuPAGE® Novex® Bis-Tris gel systems (Thermo Fisher Scientific GmbH, Dreieich, Germany). Gel was then transferred to PVDF membrane (Millipore, USA), blocked, and probed with antibodies (dil 1 : 1000). Proteins were visualized by ECL chemiluminescence (Thermo Fisher Scientific GmbH). Antibodies (for total or phosphor) ERK1/2 (sc-7383) were purchased from Santa Cruz Biotechnology. Specific antibodies for phosphor p38 MAPK (Thr180/Tyr 182) and JNK (Thr183/Tyr185) were purchased from Cell Signaling Technology, Inc. USA, NFATC1 antibody from Thermo Fisher Scientific GmbH, and anti-TRAF6 antibody and c-Fos antibody from Abcam Biotechnology Company, Cambridge, UK. Quantification of Western blots was performed with ImageJ program.

### 2.9. RNA Extraction and Real-Time PCR Analysis

Total RNA was extracted from cultured cells using a single-step method of TRIzol (Thermo Fisher Scientific, Inc.) as described [23]. cDNA was synthesized from 1 *μ*g of total RNA using RevertAid H Minus First Strand cDNA Synthesis Kit (Fermentas, Thermo Fisher Scientific) according to the manufacturer's instructions. Quantitative real-time PCR was performed with Applied Biosystems 7500 Real-Time system using Fast SYBR® Green Master Mix (Applied Biosystems, California, USA) with specific primers ([Supplementary-material supplementary-material-1]). The expression of each target gene was normalized to *β-Actin* and *Hprt* mRNA expression as reference genes, using a comparative CT method ((1/(2delta-CT)) formula, where delta-CT is the difference between CT-target and CT-reference) with Microsoft Excel 2007®.

### 2.10. Statistical Analysis

All values were expressed as mean ± SD (standard deviation) of at least three independent experiments. The power calculation was performed for 2 samples using unpaired Student's *T*-test (2-tailed) assuming equal variation in the two groups. Differences were considered statistically significant at ^*∗*^*P* < 0.05.

## 3. Results

### 3.1. Effect of 5′-HA on Cell Viability of RANKL-Induced BM Cells

**Figure 1:**
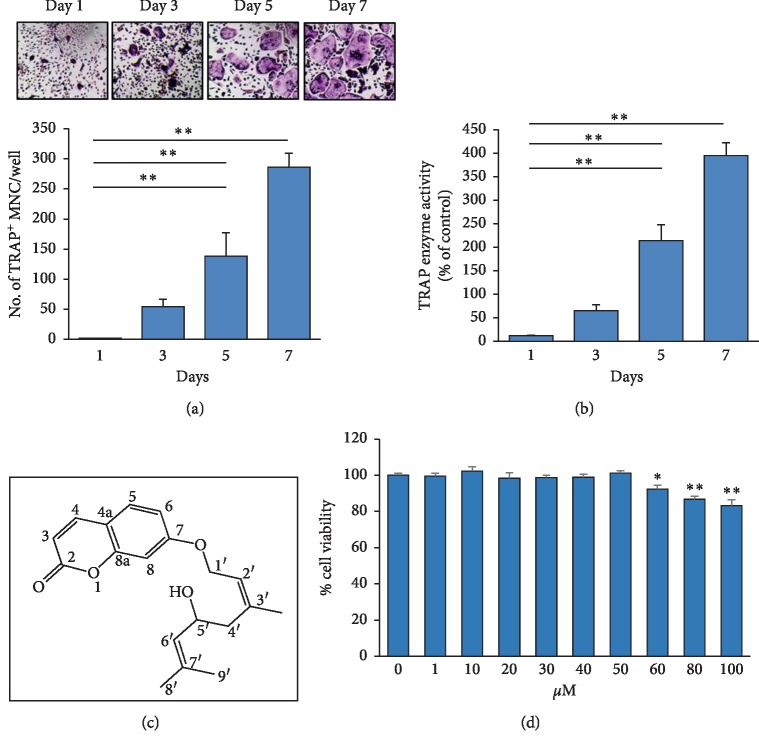
Cytotoxicity of 5′-HA on RANKL-induced osteoclastogenesis of BM cells. (a) Osteoclast differentiation of mouse BM cells. BM cells (mononuclear cells/macrophages) were induced to differentiate into osteoclasts with RANKL and M-CSF as described in Section 2. The TRACP^+^ cells with more than three nuclei per cell were counted as multinucleated cells (MNCs) at different time points. Images of TRAP staining for MNCs were shown. (b) Quantitative TRAP activity at different time points during osteoclast differentiation of BM cells. (c) Cytotoxicity of 5′-HA on RANKL-induced BM cells. Cells were induced with RANKL and M-CSF in the absence (0) or the presence of different concentrations of 5′-HA and cell viability was measured by CellTiter-Blue® cell viability assay after 3 days in culture. Values are mean ± SD of three independent experiments (^*∗*^*P* < 0.05, ^*∗∗*^*P* < 0.005 compared to day 1 for (a) and (b) and compared to RANKL-induced cells without 5′-HA for (c)).

To examine the effect of 5′-HA on osteoclastogenesis, we first established an osteoclast differentiation time point course for primary isolated murine BM cells. BM cells treated with M‐CSF and RANKL displayed the formation of multinucleated osteoclasts (with more than 3 nuclei) in association with increasing TRAP enzyme activity in osteoclasts after 7 days of treatment (Figures 1(a) and 1(b)). We further studied the cytotoxicity of newly isolated 5′-HA compound (Figure 1(c)) on osteoclasts, by measuring cell viability of BM cells in the presence of M‐CSF and RANKL with and without different concentrations of 5′-HA (1–100 *μ*M) after 3 days in culture.  Figure 1(d) shows that the toxic effect of 5′-HA started at the concentration above 50 *μ*M. Thus, we used 5′-HA between 1 and 50 *μ*M concentrations throughout this study.

### 3.2. 5′-HA Suppresses Osteoclast Differentiation

**Figure 2:**
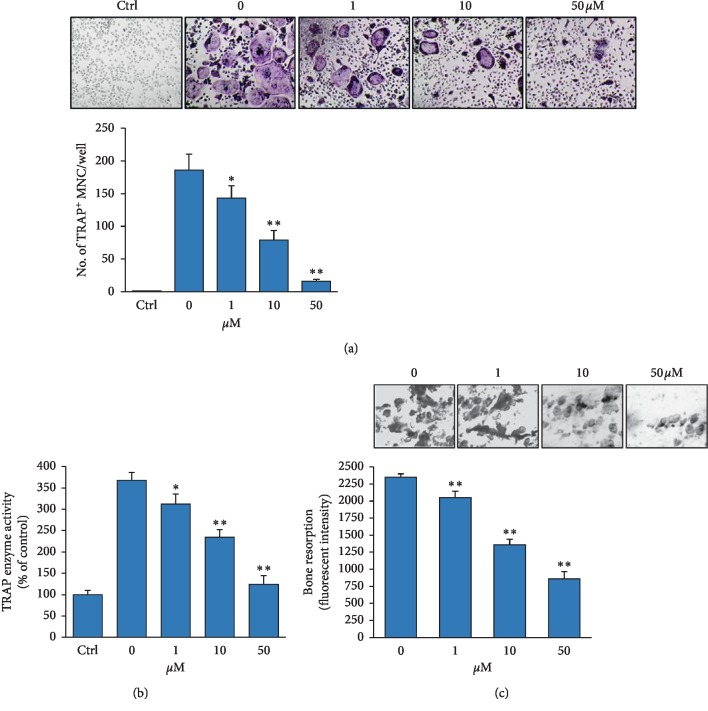
Inhibitory effect of 5′-HA on RANKL-induced osteoclastogenesis in BM cells. (a) Dose-dependent inhibitory effect of 5′-HA on osteoclast differentiation of BM cells as measured by quantification of the total number of TRACP^+^MNCs and (b) quantitative TRAP activity. BM cells were induced to differentiate into osteoclasts without (Ctrl) or with RANKL and M-CSF in the absence (0) or the presence of different concentrations of 5′-HA for 7 days. Images of TRAP staining for MNCs were shown. (c) Dose-dependent inhibitory effect of 5′-HA on osteoclast bone resorption. BM cells were induced with RANKL in the absence or the presence of 5′-HA on fluoresceinated calcium phosphate-coated 24-well plates for 6 days as described in Section 2. Images of pit resorption assay were shown. Values are mean ± SD of three independent experiments (^*∗*^*P* < 0.05, ^*∗∗*^*P* < 0.005 compared to RANKL-induced cells without 5′-HA).

We studied the effect of 5′-HA on osteoclast differentiation of murine BM cells. Addition of 5′-HA to RANKL-induced BM cells showed to exert dose-dependent inhibitory effect on a number of TRAP^+^MNCs (Figure 2(a)), as well as on TRAP enzyme activity (Figure 2(b)) during osteoclasts differentiation. Furthermore, 5′-HA exerted a dose-dependent inhibitory effect on osteoclast activity as measured by *in vitro* bone resorption assay in RANKL-induced BM cells (Figure 2(c)). These data demonstrated the inhibitory effect of 5′-HA on osteoclast differentiation and activity.

### 3.3. 5′-HA Inhibits NF-*κ*B and c-Fos/NFATc1 Activation in RANKL-Induced BM Cells

**Figure 3:**
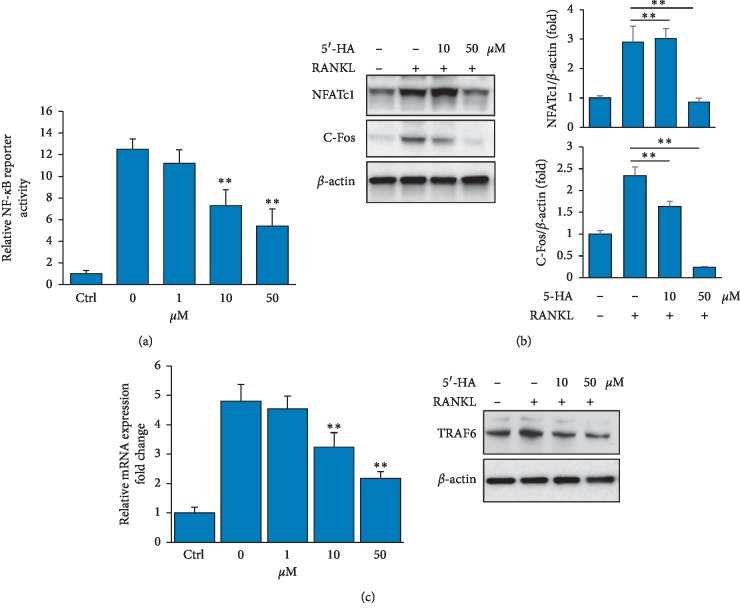
5′-HA inhibits RANKL-induced NF-kB and c-Fos/NFATc1 pathway. (a) 5′-HA inhibits RANKL-induced NF-*κ*B signaling activity. BM cells were transfected with Cignal NF-*κ*B reporter negative control or positive control and without (Ctrl) or with RANKL and M-CSF in the absence (0) or the presence of different concentrations of 5′-HA for 24 hours. Reporter activity was represented after normalization to the internal Renilla reporter. (b) Western blot analysis of C-Fos and NFATc1 protein expression. Cells were pretreated with 5′-HA (50 *μ*M) for 1 h and then stimulated with RANKL for 20 min. (c) qPCR and Western blot analysis of TRAF6 expression in RANKL-induced BM cells in the absence and the presence of different concentrations of 5′-HA. Values are mean ± SD of three independent experiments (^*∗*^*P* < 0.05, ^*∗∗*^*P* < 0.005, as compared to RANKL-induced cells without 5′-HA).

Since, the binding of RANKL to its receptor RANK stimulates the osteoclast differentiation *in vitro* via the activation of NF-*κ*B signaling pathway, we examined the effect of 5′-HA on RANKL-induced NF-*κ*B reporter activity [24, 25]. As shown in Figure 3(a), 5′-HA significantly suppressed the NF-*κ*B reporter luciferase activity in a dose-dependent manner (Figure 3(a)). We further examined the effect of 5′-HA on the protein expression of c-Fos and NFATc1, two master regulators of osteoclastogenesis. Our results demonstrated the dose-dependent inhibitory effect of 5′-HA on RANKL-induced c-Fos/NFATc1 protein expression in BM cells as assessed by Western blot analysis (Figure 3(b)). In addition, qPCR and Western blot analysis showed a significant inhibitory effect of 5′-HA on the expression TRAF6 (an upstream molecule of c-Fos/NFATc1) at both mRNA and protein expression levels in RANKL-induced BM cells.

### 3.4. 5′-HA Exerts Dose-Dependent Inhibitory Effect on mRNA Expression of Osteoclast Specific Genes

**Figure 4:**
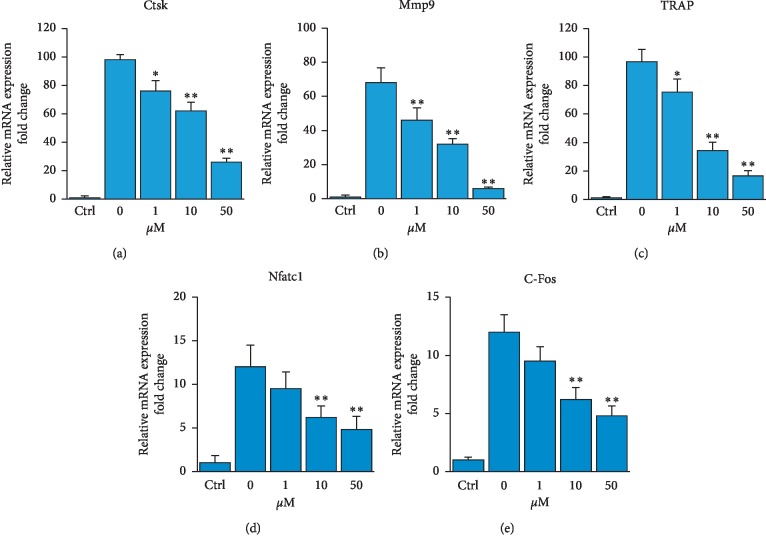
5′-HA downregulates the RANKL-induced osteoclast specific gene expression. (a–e) qPCR analysis of osteoclastic gene expression in RANKL-induced BM cells. BM cells were induced to differentiate into osteoclasts without (Ctrl) or with RANKL and M-CSF in the absence (0) or the presence of different concentrations of 5′-HA for 7 days. Each target gene was normalized to reference genes and represented as fold change over noninduced control cells. Values are mean ± SD of three independent experiments (^*∗*^*P* < 0.05, ^*∗∗*^*P* < 0.005, as compared to RANKL-induced cells without 5′-HA).

The activation of c-Fos/NFATc1 by RANKL was shown to upregulate a number of genes involved in osteoclast differentiation including *Ctsk*, *Trap,* and *Mmp9* [25, 26]. Thus, we studied the effect of 5′-HA on the mRNA expression of the above osteoclastic genes in BM cells treated with RANKL. Interestingly, 5′-HA significantly downregulated the mRNA expression of RANKL-induced *Nfatc1* and *c-Fos* genes and their target genes, *Ctsk*, *Trap,* and *Mmp9* in a dose-dependent manner as quantified by qPCR (Figures 4(a)–4(e)).

### 3.5. 5′-HA Suppresses the RANKL-Induced MAPK Pathway Activation

**Figure 5:**
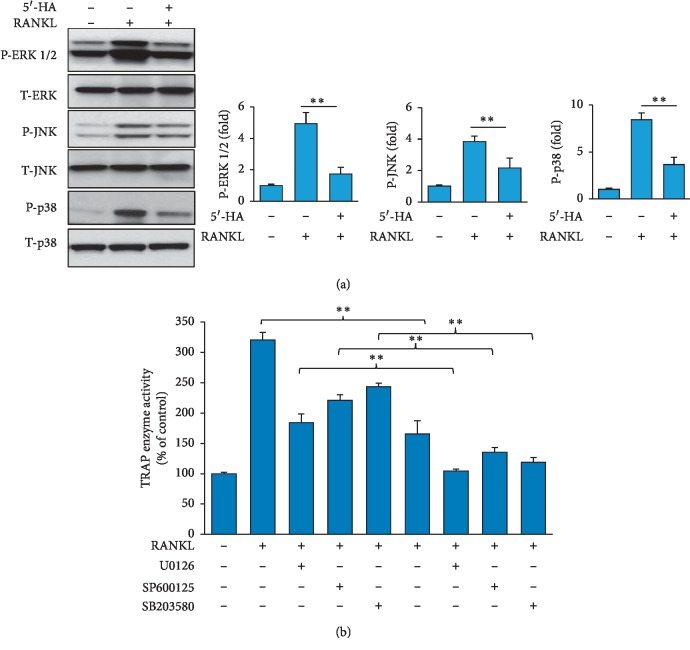
5′-HA inhibits RANKL-induced MAPKs signaling pathway in BM cells. (a) Western blot analysis of ERK1/2, JNK, and p-38 phosphorylation in RANKL-induced BM cells in the absence and the presence of 5′-HA. Cells were pretreated with 5′-HA (50 *μ*M) for 1 h and then stimulated with RANKL for 20 min. (b) Effect of blocking MAPKs on 5′-HA-inhibited osteoclastogenesis. BM cells were pretreated with specific inhibitors for ERK1/2 (U0126, 10 *μ*M), JNK (SP600125, 10 *μ*M), and p38 (SB203580, 10 *μ*M) and then induced with RANKL and M-CSF in the absence or the presence of 5′-HA. Quantitative TRAP activity was measured after 7 days of treatment and values were presented as percentage of control noninduced. Values are mean ± SD of three independent experiments (^*∗*^*P* < 0.05, ^*∗∗*^*P* < 0.005, as compared to RANKL-induced cells without 5′-HA).

We further examined the effect of 5′-HA on RANKL-induced MAPK activation [27]. As shown in Figure 5(a), treatment of BM cells with RANKL for 20 min induced ERK, JNK, and p38 phosphorylation levels, the 3 major subfamilies of MAPKs signaling pathway as assessed by Western blot analysis. Interestingly, 5′-HA significantly suppressed the RANKL-induced ERK, JNK, and p38 activation by 65.3%, 43.2%, and 57.1%, respectively. To examine the involvement of MAPK pathway in mediating the inhibitory effect of 5′-HA on osteoclast differentiation, we measured the effect of blocking ERK, JNK, and p38 activation (by the specific inhibitors, U0126, SP600125, and SB203580, respectively) on RANKL-induced osteoclastogenesis in the absence and the presence of 5′-HA using TRAP enzyme activity. As shown in Figure 5(b), blocking of ERK, JNK, and p38 activation in the absence of 5′-HA, inhibited the RANKL-induced osteoclastogenesis by 42.5%, 31.4%, and 24.1%, respectively, while synergized the inhibitory effect of 5′-HA on osteoclastogenesis by 67.5%, 57.8%, and 63.1% respectively (Figure 5(b)). Thus, the inhibitory effect of 5′-HA on osteoclastogenesis is mediated partially via suppressing the MAPK pathway.

## 4. Discussion

Increased bone resorption by osteoclast is a characteristic feature of bone loss related diseases including osteoporosis, Paget's disease of bone, periodontal disease, rheumatoid arthritis, and cancer-associated bone disease [28, 29]. In this study, we identified coumarin derivative, 5′-HA, as a novel phytochemical inhibitor of RANKL-induced osteoclast differentiation of BM cells via suppressing c-Fos/NFATc1 and MAPKs signaling pathways.

Our data demonstrated the inhibitory effect of 5′-HA on RANKL-induced osteoclast differentiation of BM cells by significantly reducing the number of TRAP^+^MNCs, TRAP activity, and NF-*κ*B signaling pathway. Consistently, several naturally occurring coumarin compounds showed antiosteoclastic bone resorption effects *in vitro* and *in vivo*. These include aesculin, psoralidin, daphnetin, bakuchiol, and esculetin [16, 19, 30, 31]. Thus, coumarin derivatives can be used as promising natural pharmaceutical agents for the treatment of osteoporosis.

After binding of RANKL to its RANK receptor on osteoclast precursors, RANKL transmits its osteoclast differentiation signals through NF-*κ*B pathway and its mediator MAPK proteins, subsequently upregulating the expression of the transcription factors c-Fos and NFATc1 to stimulate the formation and activation of osteoclasts [6, 25]. RANKL was shown to activate all three MAPK family members ERK, JNK, and p38 in association with the stimulation of osteoclast differentiation [27, 28]. In this context, our data demonstrated that 5′-HA inhibits the RANKL-induced activation of NF-kB and MAPK subfamily including p38, JNK, and ERK. Similarly, coumarin derivatives, psoralidin and esculetin, were reported to inhibit RANKL-induced osteoclastogenesis by suppressing the activation of p38, JNK, and ERK [17, 19].

Our data demonstrated that the inhibitory effect of 5′-HA on osteoclastogenesis is mediated via downregulating the expression of both osteoclastic transcription factors, c-Fos and NFATc1. The c-Fos/NFATc1 pathway plays a vital role in osteoclast formation. NFATc1 upregulates the expression of several osteoclast specific genes [32]. c-Fos belongs to the Fos gene family, which is a component of the transcriptional activator, activator protein-1 (AP-1). BM cells lacking c-Fos were unable to differentiate into mature osteoclast and mice deficient in c-Fos developed osteopetrosis, suggesting the crucial role of c-Fos in osteoclast determination [33, 34]. NFATc1 is another osteoclastic transcription factor that is stimulated and activated by RANKL to provoke the intracellular signal cascades essential for regulating terminal osteoclast differentiation. Ectopic expression of NFATc1 in osteoclast precursor cells enhanced their osteoclastogenesis even in the absence of RANKL stimulation, and mice deficient in NFATc1 expression displayed osteopetrotic defect [35, 36]. In support of our findings, coumarin derivatives including daphnetin, psoralidin, and esculetin were reported to suppress osteoclastogenesis *in vitro* through direct inhibition of c-Fos and NFATc1 expression [16, 17, 30].

Coumarin derivatives have interesting pharmacological properties, and coumarin ring system has been used as a scaffold for developing therapeutic drugs for chronic diseases [37, 38]. There are no specific receptors for natural coumarin and its synthetic analogs. However, coumarin was reported as a potential platform for designing ligands for adenosine receptors. The affinity and binding activity of coumarin toward adenosine receptors were based on the nature of its substituents [39]. Interestingly, coumarin-based selective estrogen receptor modulators (SERMs) with high affinity for estrogen receptor (ER) were synthesized and used to block osteoclastogenesis [40] and to act as an antiestrogen on breast cancer cells [41]. Thus, it is plausible that the regulatory effect of 5′-HA on osteoclast differentiation is mediated at least in part by ER on BM cells; however, this notion needs further extensive experimental work.

Taken together, our data provide 5′-HA as a novel coumarin derivative with antiosteoclastic effect that can be used for the treatment of bone resorption-related disease. However, further *in vivo* study is required to demonstrate the therapeutic effect of 5′-HA on bone resorption in osteoporotic mouse model.

## 5. Conclusion

Our data demonstrated the inhibitory effect of 5′-HA on RANKL-induced osteoclast differentiation of BM cells. The inhibitory effect of 5′-HA on osteoclastogenesis was found to be mediated by suppressing MAPK subfamily including p38, JNK, ERK, and osteoclastic transcription factors, c-Fos and NFATc1.

## Data Availability

The data used to support the findings of the study are available from the corresponding author upon request.

## Abbreviations

5′-HA: 5′-Hydroxy auraptene
BM: Bone marrow
TRAP: Tartrate-resistant acid phosphatase
MNCs: Multinucleated cells
RANKL: Receptor activator of nuclear factor kappa-Β ligand
NF-*κ*B: Nuclear factor kappa-Β
NFATc1: Nuclear factor of activated T cells, cytoplasmic 1
MCS-F: Macrophage colony stimulating factor
TNF: Tumor necrosis factor
*Mmps*: Matrix metalloproteinase
*Ctsk*: Cathepsin K.

## Supplementary Materials

Supplementary Materials Table S1: the primer sequences of all genes used by real-time PCR.
