# Supplementary material for: The Coumarin Derivative 5′-Hydroxy Auraptene Suppresses Osteoclast Differentiation via Inhibiting MAPK and c-Fos/NFATc1 Pathways
Source: Biomed Res Int. 2019 Dec 28;2019:9395146. doi: 10.1155/2019/9395146 (PMC6949687; doi:10.1155/2019/9395146)
Supplement: Supplementary Materials — Table S1: the primer sequences of all genes used by real-time PCR. [file 9395146.f1.pdf]

**Table S1. Primer sequences used for real-time RT-PCR analysis.**

| Gene name | Primer sequence (5′-3′) forward | Primer sequence (5′-3′) reverse |
|---|---|---|
| *c-Fos* | 5′-CTGGTGCAGCCCACTCTGGTC- 3′ | 5′-CTTTCAGCAGATTGGCAATCTC-3′ |
| *NFATc1* | 5′-CTCGAAAGACAGCACTGGAGCAT-3′ | 5′-CGGCTGCCTTCCGTCTCATAG-3′ |
| *TRAP* | 5′-CTGGAGTGCACGATGCCAGCGACA-3′ | 5′-TCCGTGCTCGGCGATGGACCAGA-3′ |
| *Mmp9* | 5′-CTGTCCAGACCAAGGGTACAGCCT-3′ | 5′-GTGGTATAGTGGGACACATAGTGG-3′ |
| *CtsK* | 5′-CCTCTCTTGGTGTCCATACA-3′ | 5′-ATCTCTCTGTACCCTCTGCA-3′ |
| *β-Actin* | 5′-GATATCGCTGCGCTGGTCGTC -3′ | 5′-ACGCAGCTCATTGTAGAAGGTGTGG-3′ |
| *Hprt* | 5′- TCAGTCAACGGGGGACATAAA-3′ | 5′- GGGGCTGTACTGCTTAACCAG-3′ |
